# On-farm risk factors associated with *Leptospira* shedding in New Zealand dairy cattle

**DOI:** 10.1017/S095026882000103X

**Published:** 2020-05-18

**Authors:** Y. Yupiana, E. Vallée, P. Wilson, J. F. Weston, J. Benschop, J. Collins-Emerson, C. Heuer

**Affiliations:** 1 School of Veterinary Science, Massey University, Palmerston North, New Zealand; 2 Ministry of Agriculture, Jakarta, Indonesia

**Keywords:** Dairy cattle, *Leptospira* shedding, New Zealand, on-farm, risk factors

## Abstract

This study aimed to evaluate risk factors associated with shedding of pathogenic *Leptospira* species in urine at animal and herd levels. In total, 200 dairy farms were randomly selected from the DairyNZ database. Urine samples were taken from 20 lactating, clinically normal cows in each herd between January and April 2016 and tested by real-time polymerase chain reaction (PCR) using *gyrB* as the target gene. Overall, 26.5% of 200 farms had at least one PCR positive cow and 2.4% of 4000 cows were shedding *Leptospira* in the urine. Using a questionnaire, information about risk factors at cow and farm level was collected via face-to-face interviews with farm owners and managers. Animals on all but one farm had been vaccinated against Hardjo and Pomona and cows on 54 of 200 (27%) farms had also been vaccinated against Copenhageni in at least one age group (calves, heifers and cows). Associations found to be statistically significant in univariate analysis (at *P* < 0.2) were assessed by multivariable logistic regression. Factors associated with shedding included cattle age (Odds ratio (OR) 0.82, 95% CI 0.71–0.95), keeping sheep (OR 5.57, 95% confidence interval (CI) 1.46–21.25) or dogs (OR 1.45, 95% CI 1.07–1.97) and managing milking cows in a single as opposed to multiple groups (OR 0.45, 95% CI 0.20–0.99). We conclude that younger cattle were more likely to be shedding *Leptospira* than older cattle and that the presence of sheep and dogs was associated with an increased risk of shedding in cows. Larger herds were at higher risk of having *Leptospira* shedders. However, none of the environmental risk factors that were assessed (e.g. access to standing water, drinking-water source), or wildlife abundance on-farm, or pasture were associated with shedding, possibly due to low statistical power, given the low overall shedding rate.

## Introduction

Leptospirosis is one of the most widespread bacterial diseases caused by approximately 250 serovars of pathogenic *Leptospira* spp [1]. Both animals and humans can be infected by *Leptospira*. Infected animals can shed *Leptospira* into the environment intermittently via urine for up to 40 weeks after initial detection [1, 2]. Transmission between animals and from animals to humans can occur through direct contact with infected urine or indirectly through contamination of the environment, via open wounds or the mucous membranes of eyes, nose and mouth [1].

Before the introduction of extensive vaccination with bivalent (Hardjo and Pomona) vaccines in New Zealand dairy herds in the 1980s, human leptospirosis cases in dairy farm workers related to those serovars were commonplace with an average annual incidence of 1100 notified cases per 100 000 of the resident population in 1970–1979 [3, 4]. During that period, clinical leptospirosis with leptospiruria in cattle was frequently diagnosed and largely associated with Pomona infection [5, 6]. However, *Leptospira* shedding was also identified in subclinically infected cattle [5, 7, 8]. Carter *et al*. [5] and Cordes *et al*. [7] found 0.7% and 0.4% of dairy cows, respectively, in Waikato farms were shedding *Leptospira* without showing any clinical signs. The true percentage of cattle shedding *Leptospira* might have been higher since the detection was based on microscopy and culture techniques. Current molecular techniques have higher sensitivities especially at the acute phase [9]. A small pilot study in 2011 involving 44 vaccinated dairy herds showed *Leptospira* shedding, detected by quantitative polymerase chain reaction (qPCR) and/or dark-field microscopy in 4% of 445 vaccinated dairy cows and in 30% of herds [10]. However, there were no data collected on the infecting serovar/s.

That preliminary study prompted a nationwide survey of dairy herds conducted from 2015 to 2016 which found a similar animal- and herd-level shedding prevalence. This study identified that of five serovars tested, Tarassovi was the only one positively associated with shedding [11]. A recent review of the epidemiology of notified human leptospirosis cases in New Zealand from 1999 to 2016 found Tarassovi to be the second most frequent serovar infecting dairy farmworkers after Hardjo [12]. In the Waikato region, a high-density dairy farming area, Tarassovi was the dominant serovar in notified cases of leptospirosis in dairy farm workers [13].

Investigation of risk factors for *Leptospira* exposure in dairy farm workers and people with dairy contact was conducted in New Zealand more than 30 years ago. A cross-sectional study in the Manawatu region [14] and a wider study involving the Waikato, Manawatu, Northland, Bay of Plenty and Wairarapa regions [15] showed increased time spent in the dairy shed, wearing shorts during milking, keeping pigs for sale, male gender, a previous history of leptospirosis in farmworkers, a known clinical history of leptospirosis in cattle, increased size of the milking herd and no vaccination of the herd against leptospirosis, as being risk factors associated with seropositivity to *Leptospira* in workers. However, these associations were analysed without adjustment for confounding.

While there have been no studies investigating risk factors for *Leptospira* infection in dairy cattle in New Zealand, studies in other countries suggested several risk factors. These included large herd size [16–18], the presence of other animals such as sheep, goats, swine, dogs and rodents on farm [19–22], the purchase or introduction of cattle [20, 23], increasing age of cattle [22] and surface water for drinking [16].

Based on the recent research and human notified case data as above, the serovar distribution in New Zealand dairy cattle appears to have changed since studies in the 70s and 80s and the adoption of vaccination. This supported the need to re-evaluate risk factors associated with shedding of *Leptospira* in dairy herds to better inform current measures to control *Leptospira* in dairy herds and consequently reduce exposure to workers. Therefore, this study aimed to identify herd- and cow-level risk factors associated with *Leptospira* shedding in dairy cattle farms in New Zealand.

## Methods

### Study design

A cross-sectional study of *Leptospira* shedding in urine and seroprevalence in dairy cattle in New Zealand was conducted from 5 January to 26 April 2016, primarily to evaluate the effectiveness of vaccination programmes for reducing *Leptospira* shedding, but also to re-evaluate the epidemiology of *Leptospira* on dairy farms. This study is part of a larger project and full details of the project including sample size calculation, farm recruitment, sample and data collection and laboratory testing are presented in Yupiana *et al*. [11]. Briefly, 20 adult cows from 200 randomly selected dairy herds, stratified by herd size and region throughout New Zealand, were urine sampled by the farm's veterinary service provider. This study focused on urine shedding only. Urine samples were analysed by qPCR using *gyrB* as a target gene for *Leptospira* DNA as described by Subharat *et al*. [24] and Fang *et al*. [25]. Manipulations performed on animals were approved by the Massey University Animal Ethics Committee, protocol 15/57.

### Farm data collection

Information on possible risk factors was collected using a pre-tested questionnaire (Supplementary Material 1). Data collected included general and farm demographic information, vaccination practices including vaccine/s used and vaccination protocol and timing, herd size categorised as 0–270, 271–462, 463–592 and >592 lactating cows, the environment including drinking-water sources, access to standing or floodwater and wildlife abundance, the presence of pigs, sheep, deer, dogs, whether leptospirosis had occurred in farmworkers and whether clinical leptospirosis was recently detected in animals. The questionnaires were completed at the time of sample collection by the veterinary service provider by face-to-face interview.

The information from the questionnaires was manually entered into a Microsoft Access database.

### Statistical analysis

All statistical analyses were done using R version 3.3.2 (2016-10-31). Statistical significance was accepted at *P* < 0.05.

The relationship between herd- and cow-level putative risk factors associated with the urine PCR result (positive or negative) was analysed at the individual animal level using logistic regression with a random effect for farm to adjust for unmeasured confounders at herd level and for correlation of the response within the herd.

Continuous predictors of shedding were checked for collinearity. If the Pearson's correlation coefficient between two continuous variables was greater than 0.9, only one of the two variables was retained. The decision was based on biological plausibility and the strength of the crude association with the outcome [26]. The linearity assumption for continuous predictors was tested by exploring the nature of the relationship between a continuous predictor and the outcome. If linearity was not a reasonable assumption, the variable was split into categories and factorised. The likelihood ratio test (LRT) was used to decide whether a variable or factor was significant in the model. A preferred model was determined by the lowest AIC (Akaike information criterion) [26]. The relationship between herd- and individual-level putative risk factors and the outcome were analysed in three steps. Firstly, the odds ratio for each variable was screened individually. Secondly, variables with a *P*-value of 0.2 or below were included to develop the final multivariable model by backward elimination. In this step, variables with *P*-value >0.05 were excluded from the final model. Finally, initially, non-significant variables were again added one by one to the final model to check if any of them had initially been confounded to non-significance. The criterion for retention was based on the statistical significance of the predictor [26]. Confounding was evaluated by assessing the change in a coefficient or its standard error by more than 20% before and after removing a suspected confounder from the model [26]. Biologically plausible interaction terms among all the variables in the final model were tested [26]. We calculated odds ratios by exponentiating the regression coefficients and the endpoints of their 95% confidence intervals.

## Results

### Descriptive statistics

In total, 200 dairy farms participated in the study; 65% (*n* = 130) were in the North Island and 35% (*n* = 70) in the South Island. The mean herd size was 462 milking cows (range 130–2201). This is about 10% higher than the average herd size of 419 in New Zealand in 2015/2016 [27]. The median age of sampled cows was 4 years, with a range of 2–16 years. In total, 68% of participating farms introduced replacement cows into their herds within the previous 5 years. Other animals kept on surveyed farms included sheep (33%), beef cattle (32%), pigs (20.5%) and dogs (76%). Wildlife such as rats, mice and possums was seen on 24.5% of the farms. The proportion of farmers who often saw rats (*P* = 0.02) and mice (*P* = 0.008) around milking sheds was significantly higher in the North Island than in the South Island. Troughs were the only permanent water sources for cows on 71.5% of farms, but on the remainder, cows could also access ponds, streams, valley dams and/or ditches.

In total, 94 cows (2.4%) from 53 herds (26.5%) were urine qPCR positive. There was no significant difference in *Leptospira* shedding prevalence between the North Island (27.7%) and the South Island (24.3%).

All but one farmer had conducted vaccination against serovars Hardjo and Pomona, and 54 of 199 (27%) farmers additionally vaccinated at least one age group against serovar Copenhageni. Overall, 81% vaccinated calves with at least two injections 4 weeks apart by the age of 6 months and 93% gave an annual booster to milking cows at dry-off. The only unvaccinated farm in this study was included in the statistical analysis.

### Factors associated with Leptospira shedding

#### Univariate analysis

Table 1 shows one cow-level and eight herd-level risk factors that were unconditionally associated with shedding at *P* < 0.2. At the animal level, there was a significant negative linear relationship between age and shedding. At the herd level, significant variables associated with shedding were region, breed of cow, keeping sheep or dogs on the farm, herd size (higher in large herds) and vaccine type used in heifers and adult cows. Managing cows as a single mob as opposed to multiple mobs was significantly associated with a lower shedding risk. The effect of the region was only marginally significant, but herds in Northland, Bay of Plenty and West Coast regions had higher shedding levels than Taranaki. Similarly, Friesian-Jersey crossbred cows appeared to be more prone to shedding while breed overall was only marginally significant. No other risk factors from the questionnaire were associated with shedding.

**Table 1.** Unconditional associations between potential risk factors and *Leptospira* shedding status (*P*-value <0.2)

Risk factor	Level of observation	No. of herds or cows	Odds Ratio	95% confidence interval		*P*-value
				Lower	Upper	
Region						
Taranaki	Herd	26	Ref.			
Northland		18	6.58	1.36	31.83	
North Waikato		25	4.35	0.95	19.85	
South Waikato		25	3.49	0.75	16.18	
Bay of Plenty		7	7.30	1.03	51.78	
Lower North Island		29	1.79	0.37	8.64	
West Coast		9	6.99	1.12	43.70	
Canterbury/North Otago		33	1.54	0.32	7.36	
Southland		28	2.80	0.45	17.49	0.085
Breed						
Friesian	Herd	1520	Ref.			
Friesian-Jersey		1460	2.22	1.03	4.77	
Jersey		580	0.97	0.32	2.92	
Other		140	0.46	0.04	5.30	0.105
Are all milking cows on the property managed as one mob/group						
No	Herd	56	Ref.			
Yes		136	0.36	0.18	0.74	0.005
Keep sheep on the farm						
No	Herd	137	Ref.			
Yes		63	2.34	1.15	4.77	0.019
Sizes of the milking herd						
0–270	Herd	50	Ref.			
>270–462		67	1.09	0.41	2.87	
>462–592		33	3.47	1.24	9.73	
>592		50	2.20	0.83	5.81	0.041
Age of milking cows						
Cont.	Cow	3360	0.82	0.73	0.93	0.001
Vaccine type for heifers						
2 way	Herd	111	Ref.			
3 way		51	2.25	0.98	5.17	0.055
Vaccine type for cows						
2 way	Herd	111	Ref.			
3 way		43	1.95	0.88	4.31	0.098
Number of dogs kept on the farm						
Cont.	Herd	200	1.12	0.96	1.30	0.147

#### Multivariate analysis

One animal-level and three herd-level risk factors remained in the final model (Table 2). Older cows were less likely to shed with the odds decreasing by 18% for every additional year of age. Keeping sheep with no dogs on farms increased the odds of cows shedding *Leptospira* (OR 5.57, 95% CI 1.46–21.25), additional dogs with no sheep kept on the farm increased the odds of shedding (OR 1.45, 95% CI 1.07–1.97), having both sheep and dogs on the farm increased the risk of shedding and managing milking cows in a single rather than multiple groups reduced the odds of a cow shedding (OR 0.45, 95% CI 0.2–0.99).

**Table 2.** Final logistic regression model with a random effect for herd showing associations between *Leptospira* shedding status and potential risk factors

Risk factor	Coef.	Odds ratio	95% confidence interval		*P*-value
Age of milking cows (years)					
Continuous	−0.19	0.82	0.71	0.95	0.007
Sheep on the farm					
No (ref.)					
Yes	1.72	5.57	1.46	21.25	0.012
No. of dogs on the farm					
Continuous	0.37	1.45	1.07	1.97	0.016
Sheep on the farm × number of dogs on the farm					
Continuous	−0.41				0.036
Are all milking cows on the property managed as one group					
No (ref.)					
Yes	−0.79	0.45	0.2	0.99	0.049

## Discussion

This is the first report describing risk factors for *Leptospira* shedding in dairy cows in New Zealand. This analysis was prompted by the observation that cows in 26.5% of dairy herds shed *Leptospira* in urine [11]. The study [11] showed serological evidence for Tarassovi, not available in *Leptospira* vaccines in New Zealand, in 75% of herds and 17% of cows. Theoretically, the shedding might be due to other non-vaccine serovars (Copenhageni and Ballum). However, this is unlikely due to the lack of an association between serology and urine PCR. Shedding due to vaccine serovars Hardjo and Pomona is highly unlikely because again, they were not associated with shedding and vaccination against these serovars was deemed to be efficacious [28].

This paper was intended to provide a better understanding of the risk profile of cows and herds with respect to shedding. Thus, we hypothesised that factors other than vaccination would explain the shedding rates. Of particular interest were putative infection sources such as drinking-water sources, access to standing water on pasture, rivers, valley dams or floodwater and exposure to wildlife or other domestic animals. The risk factors identified were younger age, larger herds and the presence of sheep or dogs on farms, though the risk appeared to be less when both sheep and dogs were present on-farm.

Few recorded potential risk factors were significant in the final regression model. One explanation is the low prevalence of shedding (2.4%) in cows, resulting in low statistical power for logistic regression analysis. The absence of statistical significance is therefore poor evidence that non-significant potential factors pose no risk. For example, the risk associated with exposure to water and wildlife, which are biologically plausible, might well be undetectable using the approach employed here. Hence, while the survey was appropriate to identify a larger number of risk factors had the shedding prevalence been higher, in the event, identification of risk factors was constrained by low power. Nevertheless, there were a few factors that the study was able to identify.

The risk of shedding linearly decreased with cow age from 2 to 6+ years. This may be a function of exposure time. If exposure is more or less constant, older cattle would be exposed repeatedly and be expected to develop a stronger cell-mediated immune response (CMI) over time [29]. Consistent with our finding, a study in Waikato [7] has shown 69% of shedders (*vs.* 31% in the population) were 2- and 3-year-olds and the other 31% ranged from 4 to 9-years-old. Another study suggested that heifers were infected after their introduction into the milking herd [30]. The authors reported a lower proportion of clinical cases in cows having had four or more lactations than in younger cows, supporting an age effect in the epidemiology of infection and disease.

The finding that younger cows were more likely to shed than older cows potentially poses a higher risk of exposure for farmers and farm workers while milking first lactation heifers. In New Zealand, young cattle are often grazed away from the farm until they are old enough to enter the milking herd as heifers. As vaccination status of these animals may be uncertain they may be at greater risk of infection from the vaccinal serovars and present a risk. Heifers that were introduced to the adult herd and milked for the first time may suffer a relatively high level of stress. They may be more likely to kick the cups off and urinate [31] increasing the likelihood of exposure of workers.

Studies elsewhere suggested a relationship between the seroprevalence in cattle and the presence of cervids [20] and sheep/goat [22]. Our data demonstrated that the presence of sheep on dairy farms was a risk factor for *Leptospira* shedding in cows. *Leptospira* shedding in sheep in New Zealand was a common finding [32, 33] and sheep farmers rarely vaccinate against L*eptospira*. Fang *et al*. [32] has shown that urinary shedding and seropositive rates were 31% and 21%, respectively, in sheep and in cattle. In New Zealand, sheep are regarded to be a reservoir host for Hardjo [33]. However, antibodies to Pomona [34], Copenhageni, Ballum and Tarassovi [35] have also been detected. Mannewald *et al*. [35] recently showed 14% seroprevalence to Tarassovi in sheep. This was higher than 2.6% using the same MAT cut-point reported 30 years prior [36], suggesting a change in the epidemiology of this serovar, consistent with recent data for dairy cattle [11] and humans [12]. However, isolation of this serovar in sheep has not been reported. Thus, the role of sheep as a source of Tarassovi transmission on dairy farms cannot be confirmed without new data, but unvaccinated sheep could still be a source of infection of Hardjo and Pomona in herds where vaccination is not optimal.

An increased number of dogs on-farm was associated with an increased risk of having one or more shedding cows in the herd. Favero *et al*. [19] also found that cattle were more likely to be seropositive to *Leptospira* when dogs had access to pasture. In New Zealand, a study investigating *Leptospira* antibody against Hardjo, Pomona, Copenhageni and Ballum in dogs showed a significant association between seropositivity to Hardjo and farm working dogs as opposed to other breeds [37] suggesting a possible transmission to cows from dogs. Tarassovi was not tested in Harland's study. This serovar was isolated from pigs and dogs in New Zealand about 40 years ago [38, 39]. Hence, transmission from dogs to dairy cattle is unlikely, given the low concentration of leptospires in urine and the poor survival of *Leptospira* in dog urine [3]. Having few dogs and hundreds of cows on a typical dairy farm, it is much more plausible that leptospires are transmitted from cows to dogs, not from dogs to cows as suggested by the association in our study.

Sheep and dogs on-farm being a risk factor for shedding in cows may be a spurious association, considering the relatively small numbers of sheep (median = 6) and dogs (median = 2) kept on the farms. Unmeasured factors related to having other animals on the farms might have contributed to the higher risk of shedding in cows. For example, in our study, we found there were correlations between the presence of sheep and presence of beef cattle and/or pigs on the farms and, an increased number of dogs was also associated with the presence of cattle and/or pigs. These associations are not readily explainable.

The presence of rodents is usually associated with a contaminated environment with *Leptospira* [40] that potentially increased risk of *Leptospira* transmission to other mammals. In our data, however, an association between the presence of rodents and infection of cows could not be established. This might be because the shedding we observed in dairy cows was not related to a serovar adapted to rodents. In New Zealand, Ballum is the usual serovar identified in rodents. Previous studies reported that 28–30% of rodents were seropositive to Ballum [41, 42], but only 3% of cows in our study had this serovar and this was not related to shedding [11]. Tarassovi was rarely found in rodents [42] which again are consistent with the lack of an association between rodents and shedding in cows. A study in an urban environment in Brazil showed a significant association between the presence of rodents and seropositivity to *Leptospira* spp. including Hardjo and Icterohaemorrhagiae in cows [21]. However, since serovar/host relationships of *Leptospira* are highly specific for the country and urban *vs.* rural environments, overseas studies may bear little relevance to New Zealand. Out of all respondents, only 24.5% reported seeing rodents, which possibly reflects a low sensitivity of detection. Mice and rat species present in New Zealand can be present on pasture or around buildings, especially brown rats *Rattus norvegicus* [43] and since their activity is mostly nocturnal, they may not be seen unless an intensive control programme is in place. While the low probability of detection will likely have decreased the statistical power to detect an association, it is likely the same between positive and negative farms, so non-differential and so the point estimate (crude OR 1.53, 95% CI 0.64–3.66, data not shown) is likely unaffected.

Access to surface water has been commonly associated with *Leptospira* infection [44] but was not a significant factor in our study despite there being 29% of farms where cows had access to water sources other than troughs. A possible explanation is that Tarassovi and Hardjobovis, two of the candidates infecting serovars, belong to the *L. borgpetersenii* species and survive for relatively short periods in water [45]. Therefore, surface water might not be an important source for transmission of circulating leptospires on New Zealand dairy farms. Surface water may be a higher risk source of infection when rodents are common. A study in Brazil found a significant association between access to streams and seropositivity of animals to Hardjo/Wolffi or Icterohaemorrhagiae [16]. The authors inferred that rodents carrying Icterohaemorrhagie might have contaminated the water and that this exposed animals to the bacteria. Similarly, another study in Brazil showed a significant association between flooded pasture and seropositivity of animals to serovar Hardjo [21]. In contrast, a study of beef cattle in Ireland did not find the presence of a river as a risk factor [18]. Clearly, differences in the epidemiology of *Leptospira* infection are influenced by different environmental factors and the absence of association supports the hypothesis that most of the shedding is due to a serovar with a short environmental survival such as Tarassovi or Hardjobovis.

Large herds were more likely to harbour shedders than small herds. As for most pathogens, several studies [17, 18, 23] have shown that large herd size was associated with a higher risk of *Leptospira* transmission in cattle due to more frequent contact between infectious and susceptible animals. Large herds also have more contact with other herds through purchases and contract heifer grazing than small herds, hence are more likely to introduce shedders than small herds. Thus, larger herds are more likely to circulate and maintain the bacteria in the dairy population.

While some of the associations discussed above may be biologically plausible, a cross-sectional study such as this can only generate hypotheses about possible causal pathways. Exposure could have occurred at any time and did not necessarily precede the time of infection and shedding. For example, milking cows might have been infected before contact with sheep or dogs. Hence caution must be exercised when interpreting results from a cross-sectional study such as this. A longitudinal study design would be more appropriate to investigate the epidemiology and risk factors for shedding of the serovar/s identified in dairy cattle in New Zealand. In addition, the PCR used did not allow the identification of the shed species or serovars. Hence, results only apply to shedding in general and the identification of infecting species or serovar could possibly help to identify specific risk factors.

In summary, we conclude that younger dairy cows are more likely to shed *Leptospira* on New Zealand dairy farms. Farmworkers may use this information to take extra care and precautions when milking first calving heifers. While the presence of sheep and dogs was positively associated with shedding in cows, the biological plausibility of these species as risk factors requires further study.

## Supplementary material

For supplementary material accompanying this paper visit http://dx.doi.org/10.1017/S095026882000103X. click here to view supplementary material
