# Supplementary material for: On-farm risk factors associated with Leptospira shedding in New Zealand dairy cattle
Source: Epidemiol Infect. 2020 May 18;148:e219. doi: 10.1017/S095026882000103X (PMC7641893; doi:10.1017/S095026882000103X)
Supplement: Supplementary file 1 [file S095026882000103Xsup001.docx]

| 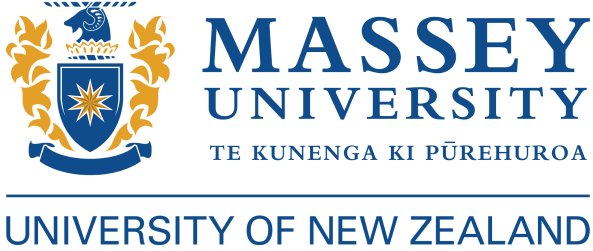 | **EpiCentre**  **Institute of Veterinary, Animal and Biomedical Sciences**  Private Bag 11 222  Palmerston North  New Zealand  Telephone:+64 (6) 350 5270.  Facsimile: +64 (6) 350 5716  [www.massey.ac.nz](http://www.massey.ac.nz) |
| --- | --- |

**A Study of Leptospirosis Vaccination in Dairy Cattle**


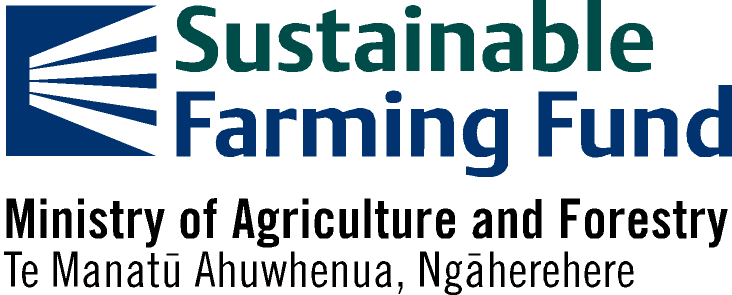

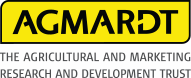

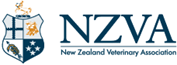


WAIRARAPA VETERINARY CLUB


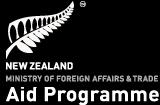

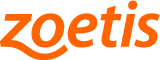

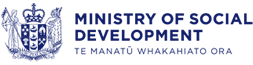

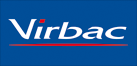


**FARMER’S CONSENT**

I have read the “Farmer Information Sheet” I received by email and understand the project and my role as a participant. Any questions have been answered to my satisfaction, and I understand that I may ask further questions at any time.

I agree to participate and will make my animals available for urine, blood and milk sampling free of charge. I am willing to provide information for the questionnaire.

YES NO

I give consent to researchers to access herd testing data for milk production and reproduction data of sampled cows during the 2014-15 season.

YES NO Not enrolled

If YES, please state the herd testing organisation (e.g. LIC): ______________________

I agree that the samples and data collected today may be used for testing for other animal diseases according to the confidentiality clause in the emailed “Farmer Information Sheet”.

YES  NO

| **Signature:** |  | | **Date:** |  |
| --- | --- | --- | --- | --- |
|  | | | | |
| **Full Name - printed** | |  | | |
|  | |  | | |

|  |
| --- |
|  |

**FARM QUESTIONNAIRE**

This questionnaire collects information about the farm, leptospirosis, vaccination practices, and other risk factors for leptospirosis in dairy herds. Data will be used in conjunction with the analysis of blood and urine from cows, and a bulk tank milk sample.

The completed questionnaire MUST be returned to Massey University along with the Farmer’s consent (above), “Sampled Animal Data Form” and samples. Please ensure that the most appropriate person on the farm is interviewed, to ensure that the most accurate data are provided.

**CONTACT DETAILS**

- 1. **Property name**: ______________________________________________________________________________
  2. **Farm address**: ­­­________________**________________________________________________________________**

**_____________________________________________________________________________________________________**

_____________________________________________District/region: _____________________________________________

- 1. **Dairy supplier number** : ______________________________________________________________________________
  2. **Contact person** **(name):** _______________________________________________________________________________

The contact person is:

Owner Manager Sharemilker Other____________________________________________

Phone & mobile: _______________________________________________________________________________________

Email: ___________________________________________________

- 1. **Person interviewed (name):** _____________________________________________________ same as above

If not the same: Owner Manager Sharemilker

Other_____________________________________________________

Phone & mobile: _______________________________________________________________________________________

Email: _______________________________________________________

- 1. **Veterinarian/Veterinary technician** (*name*): ______________________________________________________

Phone & mobile: _______________________________________________________________________________________

Email: _______________________________________________________

Veterinary Practice: ___________________________________________________________________________________

Address:______________________________________________________________________________________________________________________________________________________________________________________________________________

- 1. **Date of visit for sample collection**: ______________________________________________

**GENERAL FARM INFORMATION**

- 1. **What is the size of this farm: i.e. milking platform**? ________________________hectares (effective)
  2. **Farm management**

Owner managed Manager employed Sharemilker

Other, please state: **_____________________________________________________________________**

- 1. **How many years has the current manager been in charge of the herd**?

______________ years UNSURE

- 1. **Are all milking cows on the property managed as one mob**?

YES NO

- 1. **Calving pattern (*tick as applicable*)**:

Spring: planned start of calving (date): ________________

Autumn: planned start of calving (date): ________________

Non- seasonal calving

- 1. **Breed composition**:

________% Friesian ________% Jersey _________% Friesian-Jersey Cross

________% Other Breed UNSURE

- 1. **Numbers of dairy cattle on this farm on the day of sampling**:

| Age | Numbers | | |
| --- | --- | --- | --- |
|  | Total | On the milking platform | Away/out grazing |
| Calves (male + female, 0-12 months) |  |  |  |
| Heifers (12-24 months) |  |  |  |
| 1^st^ lactation heifers |  |  |  |
| Adult lactating cows |  |  |  |
| Bulls (12-24 months) |  |  |  |
| Bulls (24+ months) |  |  |  |

- 1. **Do you house your milking cows at any time e.g. herd home?**

YES NO

If yes: please describe:

- 1. **Has this herd been fed Palm Kernel or other concentrate feed during this season or last season?** YES NO

If **YES**, was there sign of rodent faeces ever seen on the feedstuff?

YES NO

- 1. **Have you purchased any replacement stock in the past 5 years?**

Milking cows NO YES if **YES**, how many consecutive seasons? _________

How many bulls have been purchased for breeding each year: ____________

- 1. **Have purchased animals been vaccinated against leptospirosis**?

| Heifers | Adult cows | Bulls |
| --- | --- | --- |
| All | All | All |
| Some | Some | Some |
| None | None | None |
| Unsure | Unsure | Unsure |

**VACCINATION PRACTICES**

- 1. **Have you ever vaccinated this dairy herd against leptospirosis**?

YES NO UNSURE (If **NO**, go to **Question 25**)

- 1. **If YES, for how many years have you vaccinated your dairy cattle against leptospirosis** (*tick one answer*)?

0-5 years

5-10 years

10-20 years

More than 20 years

- 1. **Describe the leptospirosis vaccination programme that has been/will be implemented for CALVES this season (2015/16).**

|  | **Age (months)** | **Calendar month(s)** | **Who administered the vaccine? *(Please tick)*** | | | **Vaccine name (see below)** |
| --- | --- | --- | --- | --- | --- | --- |
|  | | | Manager | Worker | Vet |  |
| 1^st^ vaccination |  |  |  |  |  | Unsure |
| 1^st^ booster  (*if given*) |  |  |  |  |  | Unsure |
| 2^nd^ booster  (*if given*) |  |  |  |  |  | Unsure |

*Leptavoid 2 Leptoshield Leptoshield 3 Ultravac 7 in 1 Lepto-2way Lepto-3way*

- 1. **Describe the leptospirosis vaccination programme that has been/will be implemented for HEIFERS and COWS this season (2015/16).**

|  | **Calendar month(s)** | **Who administered the vaccine? *(Please tick)*** | | | **Vaccine name (see below)** |
| --- | --- | --- | --- | --- | --- |
| **Heifers (12-24 months)** | | Manager | Worker | Vet |  |
| Vaccination |  |  |  |  | Unsure |
| 2^nd^ vaccination (if given) |  |  |  |  | Unsure |
| **Cows (24+ months)** | | Manager | Worker | Vet |  |
| Vaccination |  |  |  |  | Unsure |
| 2^nd^ vaccination (if given) |  |  |  |  | Unsure |
| **Are bulls always vaccinated at the same time as cows?  YES  NO**  If **NO**, complete this table. If **YES**, go to the next question | | | | | |
| **Bulls** | | Manager | Worker | Vet |  |
| Vaccination |  |  |  |  | Unsure |
| 2^nd^ vaccination (if given) |  |  |  |  | Unsure |

- 1. **Has your vaccination programme been the same during the past 5 years?**

YES NO UNSURE

If **YES**, go to **Question 25**

**If NO,** please complete the table in **Question 24** (*next page*)

- 1. **If NO, please explain the differences from the current season (as above).**

| Season |  | Calves (1-12 months) | Heifers, adult cows and bulls |
| --- | --- | --- | --- |
| 2015/16 | Timing |  |  |
|  | Who |  |  |
|  | Vaccine |  |  |
| 2014/15 | Timing |  |  |
|  | Who |  |  |
|  | Vaccine |  |  |
| 2013/14 | Timing |  |  |
|  | Who |  |  |
|  | Vaccine |  |  |
| 2012/13 | Timing |  |  |
|  | Who |  |  |
|  | Vaccine |  |  |
| 2011/12 | Timing |  |  |
|  | Who |  |  |
|  | Vaccine |  |  |

- 1. **Do you administer other vaccines or other whole herd treatments (such as trace elements, dry cow therapy, anthelmintic) at the same time as vaccinating against leptospirosis**?

Always Sometimes Never Unsure

- 1. **If always or sometimes, please state which:**

_____________________________________________________________________________________________

_____________________________________________________________________________________________

**OTHER ANIMALS**

- 1. **Do you keep any other domestic animal species on your property**?

YES NO If **NO**, go to **Question 32**.

- 1. **If YES, how many**?

| _______ Beef cattle | _______ Goats |
| --- | --- |
| _______ Sheep | _______ Pigs |
| _______ Deer | _______ Dogs |
| _______ Horses | _______ Cats |
| _______ other: _______________ | _______ other: _______________ |

- 1. **Do you vaccinate any of these animals against leptospirosis**?

YES NO UNSURE

- 1. **Do other species ever come in direct or indirect contact with dairy cattle**?

YES NO UNSURE

- 1. **If YES, how do they come in contact with dairy cattle?** *(please tick)*

| Other species | Grazed same paddock, same time | Alternately grazed | Share water source | Over the fence | Dairy cattle contacted | | |
| --- | --- | --- | --- | --- | --- | --- | --- |
|  |  |  |  |  | Calf | Heifer | Adult |
|  |  |  |  |  |  |  |  |
|  |  |  |  |  |  |  |  |
|  |  |  |  |  |  |  |  |
|  |  |  |  |  |  |  |  |
|  |  |  |  |  |  |  |  |
|  |  |  |  |  |  |  |  |
|  |  |  |  |  |  |  |  |
|  |  |  |  |  |  |  |  |
|  |  |  |  |  |  |  |  |

- 1. **Have you noticed any evidence of rodents or wildlife on/ near the milking platform**? (*please tick*)

| Species | Often | Sometimes | Rarely | Never |
| --- | --- | --- | --- | --- |
| Rats |  |  |  |  |
| Mice |  |  |  |  |
| Possums |  |  |  |  |
| Ferrets, stoats, weasels |  |  |  |  |
| Hedgehogs |  |  |  |  |
| Rabbits |  |  |  |  |
| Hares |  |  |  |  |
| Feral pigs |  |  |  |  |
| Feral deer |  |  |  |  |
| Feral sheep or goats |  |  |  |  |
| Feral cats |  |  |  |  |

- 1. **Rodents** are controlled by

poison trapping dogs/cats no control

- 1. **Wildlife habitat**? (*please tick as many as apply*)

Farm borders a national park, forestry or native bush

Farm has areas of bush/forestry that are fenced off

Farm has areas of bush/forestry that are not fenced off

There is no wildlife habitat other than pasture

Other? Please explain _________________________________________________

**ENVIRONMENT**

- 1. **Please describe the topography of the farming area as percentage (%) of pasture**:

     _________% Flat _________% Rolling __________% Hill

- 1. **Please describe the soil type(s)**: ___________________________________________________
  2. **Do the milking cows have access to water sources other than troughs**

YES NO

- 1. **If YES, please tick those that apply**:

Dams

Stream or river

Irrigation ditches

Natural spring(s)

Ponding of water after heavy rainfall

Other (*please specify*): ______________________________________________________

UNSURE

- 1. **Do you store milking shed effluent**?

YES NO If **No**, go to **Question 43**

- 1. **If YES, do you spray effluent on pasture**?

YES NO

- 1. **If YES, how long after spraying effluent do you graze cattle back on that pasture?**

Time from spraying to grazing to days

- 1. **Which of the following practices are followed to manage the risk of leptospirosis while milking**? (*please tick*)

Milkers wear **gloves** always sometimes never

Milkers wear **eye protection** always sometimes never

Milkers wear **overalls** always sometimes never

Milkers wear **gumboots** always sometimes never

Milkers do not **eat or smoke**. always sometimes never

**43. Do you apply other management practices to reduce the risk of leptospirosis in workers?** Please describe: _______________________________________

_____________________________________________________________________________________________

**HUMAN Leptospirosis FLU-LIKE ILLNESS**

- 1. **Has there been any flu-like illness of anyone in contact with the dairy cattle within the past 2 years?**

YES NO UNSURE

- 1. **Has there been any medical diagnosis of leptospirosis of anyone in contact with the dairy cattle within the past 2 years?**

YES NO UNSURE

**CLINICAL LEPTOSPIROSIS IN ANIMALS**

- 1. **Have there been any veterinary or laboratory diagnosed, or suspected, cases of leptospirosis in dairy cattle on your property within the past 5 years**?

YES NO UNSURE

- 1. **If YES, Please complete the table below (next page)**

| Clinical Syndrome | Number | When (month/year) | Confirmed by laboratory OR vet | Serovars (if known) |
| --- | --- | --- | --- | --- |
| Calf redwater |  |  | YES NO |  |
| Abortion |  |  | YES NO |  |
| Mastitis |  |  | YES NO |  |
| Septicaemia |  |  | YES NO |  |
| Death |  |  | YES NO |  |
| Stillbirth |  |  | YES NO |  |
| Premature birth |  |  | YES NO |  |
| Sudden drop of milk production |  |  | YES NO |  |
|  |  |  | YES NO |  |

**VACCINATION** (to be completed by the attending Veterinarian)

**From your practice records, please extract data about the Leptospirosis vaccine brand/s used on this farm up to the past 5 years if available.**

| Supply date | Name of vaccine | Number of doses | Age group |
| --- | --- | --- | --- |
|  |  |  | Calves Heifers/cows/bulls |
|  |  |  | Calves Heifers/cows/bulls |
|  |  |  | Calves Heifers/cows/bulls |
|  |  |  | Calves Heifers/cows/bulls |
|  |  |  | Calves Heifers/cows/bulls |
|  |  |  | Calves Heifers/cows/bulls |
|  |  |  | Calves Heifers/cows/bulls |
|  |  |  | Calves Heifers/cows/bulls |
|  |  |  | Calves Heifers/cows/bulls |
|  |  |  | Calves Heifers/cows/bulls |
|  |  |  | Calves Heifers/cows/bulls |
|  |  |  | Calves Heifers/cows/bulls |
|  |  |  | Calves Heifers/cows/bulls |
|  |  |  | Calves Heifers/cows/bulls |
|  |  |  | Calves Heifers/cows/bulls |
|  |  |  | Calves Heifers/cows/bulls |
|  |  |  | Calves Heifers/cows/bulls |
|  |  |  | Calves Heifers/cows/bulls |

**Thank you for completing this questionnaire**
